# Navigating Dental Implant Information on Social Media: A Qualitative Exploration of Digital Health Literacy and Resilience to Misinformation

**DOI:** 10.3390/healthcare14193298

**Published:** 2026-10-03

**Authors:** Waad Alshehri, Rayan Sharka, Mansour Alghamdi, Ammar Almarghlani

**Affiliations:** 1 Ibn Sina National College for Medical Studies, Jeddah 22421, Saudi Arabia; ms2420706@ibnsina.edu.sa; 2 Department of Oral and Maxillofacial, Faculty of Dental Medicine, Umm Al-Qura University, Makkah 24382, Saudi Arabia; mhghamdi@uqu.edu.sa; 3 Department of Periodontics, Faculty of Dentistry, King Abdulaziz University, Jeddah 21589, Saudi Arabia; mabdulhalim@kau.edu.sa

**Keywords:** dental implants, social media, misinformation, digital health literacy, qualitative research, patient decision-making, health communication

## Abstract

**Highlights:**

**What are the main findings?**

- Patients did not rely on social media alone and often checked information before making decisions.
- Dentists remained the most trusted source of information, while social media was generally viewed as a starting point rather than a basis for treatment decisions.

**What are the implications of the main findings?**

- Trust, verification, and critical thinking may help patients avoid misleading implant-related information online.
- Improving digital health literacy may support more informed decisions about dental implant treatment.

**Abstract:**

Background/Objectives: Social media has become a common source of information about dental implant treatment. While it offers easy access to treatment-related information, concerns remain about the quality and accuracy of online content. This study explored how patients evaluate and respond to dental implant information on social media and the factors and behaviors that may help them navigate potentially misleading information. Methods: A qualitative study was conducted using semi-structured interviews with adult patients attending a dental school clinic in Saudi Arabia. Participants were purposively recruited and interviewed between March and July 2026. Interviews explored social media use, perceptions of information credibility, experiences with misinformation, information verification practices, and treatment-related decision-making. Data were analyzed using Braun and Clarke’s reflexive thematic analysis. Results: Five themes were identified: (1) trust in professional and experiential sources, (2) verification before decision-making, (3) the influence of reputation and social proof, (4) skepticism toward potentially misleading information, and (5) social media as a source of information rather than a final authority. Participants commonly relied on dentists, cross-checked information across sources, drew on patient experiences and recommendations, and questioned exaggerated or commercially motivated claims before making treatment decisions. Conclusions: Participants reported using trust, verification, social influences, and critical evaluation when navigating dental implant information on social media. These behaviors may support informed treatment-related decision-making and highlight the importance of digital health literacy, as well as the role of dentists and health organizations in providing accessible and trustworthy information.

## 1. Introduction

Oral diseases remain a major public health challenge worldwide, affecting an estimated 3.5 billion people and contributing substantially to disability, reduced quality of life, and healthcare costs [1]. Tooth loss caused by dental caries, periodontal disease, trauma, and other oral conditions continues to affect millions of adults worldwide. As populations age and life expectancy increases, the demand for tooth replacement options has also grown [1]. Dental implants have become a widely accepted treatment for replacing missing teeth because of their functional and aesthetic benefits [2]. Consequently, many patients seek information about implant procedures, costs, risks, benefits, and expected outcomes before making treatment decisions [3]. The increasing use of digital technologies has transformed how patients access health information [4]. Patients increasingly use social media and other online platforms to obtain information about dental implant treatment before seeking professional care [5,6,7]. Online content may include educational material, promotional content, patient testimonials, clinical case presentations, and discussions related to implant treatment [8,9,10].

Social media has become an increasingly important source of oral health information across Arab populations and is influencing how individuals engage with dental care. Research from Saudi Arabia suggests that social media can influence oral health knowledge, treatment preferences, and healthcare-seeking behaviors, particularly among younger populations and users of visual platforms such as Instagram, TikTok, and Snapchat [11]. Studies have also shown that social media influences patients’ interest in dental treatments and may affect treatment-related decisions, particularly in esthetic dentistry [12]. Similar observations have been reported in the United Arab Emirates, where social media has been identified as an important factor in shaping trust, credibility, and patient decision-making within dental settings [13].

Despite these potential benefits, concerns remain regarding the quality and credibility of oral health information available online. Recent evidence from Saudi Arabia found that trust in social media as a source of oral health information was associated with higher levels of oral health misconceptions, whereas following dental-specific accounts was associated with fewer misconceptions [14]. Previous studies have also suggested that social media can influence attitudes, expectations, and perceptions related to dental treatment [12]. However, limited research has explored how Arab patients evaluate information about dental implant treatment encountered on social media, how they assess its credibility, or how they respond to potentially misleading information.

Although social media can improve access to information and increase patient awareness, the quality and accuracy of online content can vary considerably [6]. Patients may therefore encounter both evidence-based information and misleading claims while seeking information about dental implants, creating challenges for informed decision-making [5,8,9]. Existing research has mainly focused on assessing the quality and reliability of dental implant information on platforms such as TikTok and YouTube, often reporting wide variation in content quality and educational value [7,10,15]. In contrast, far less is known about how patients interpret, evaluate, and respond to such information when considering dental implant treatment.

Patients no longer rely solely on healthcare professionals for information and increasingly use digital resources to search for and evaluate health information before making health-related decisions [16,17]. Decisions about dental implant treatment are often complex and involve multiple considerations, including treatment procedures, surgical aspects, costs, expectations, and anticipated outcomes. Analyses of online discussions have shown that these topics are among the most frequently discussed concerns related to dental implant treatment [18,19]. Consequently, how patients evaluate and respond to information may influence treatment-related decisions.

Understanding how patients judge the credibility of online information is particularly important in dental implant treatment, where information comes from a variety of professional, commercial, and patient-generated sources [5,10,19]. Exploring how patients evaluate and respond to such information may help identify the factors that support informed decision-making and effective use of health information in the digital environment.

The widespread availability of health information on digital platforms has increased concerns about the spread of misinformation. Research has shown that health misinformation can influence beliefs, attitudes, and healthcare decision-making, while difficulties in distinguishing reliable information from misleading content remain common among social media users [20,21,22]. In response, increasing attention has been given to the concept of misinformation resilience, which refers to an individual’s ability to recognize, evaluate, and resist misleading information while making informed decisions [23].

Resilience to misinformation involves the ability to critically evaluate information and respond appropriately when exposed to misleading content [23]. Although these issues have received growing attention in health communication research, little is known about how patients navigate implant-related information encountered on social media. Therefore, this study aimed to explore how patients perceive, evaluate, and respond to information about dental implant treatment encountered on social media and identify the factors and behaviors that contribute to resilience against misinformation in this context.

## 2. Materials and Methods

### 2.1. Ethical Considerations

Ethical approval was obtained from the Institutional Research Review Board of Ibn Sina National College for Medical Studies, Jeddah, Saudi Arabia (Reference No. IRRB-09-03022026; approval date: 3 February 2026). Written informed consent was obtained from all participants prior to data collection. Participation was voluntary, and all interview data were anonymized to ensure confidentiality.

### 2.2. Study Design

This study employed an exploratory qualitative design to investigate how social media influences patients’ perceptions of dental information and its impact on trust and decision-making regarding dental treatment. Data were collected through individual semi-structured interviews, which provided participants with the flexibility to describe their experiences, beliefs, and information-seeking behaviors in their own words [24]. This approach was considered appropriate for exploring participants’ perspectives in depth and gaining a nuanced understanding of how they evaluate and respond to oral health information encountered online.

The study is reported in accordance with the Consolidated Criteria for Reporting Qualitative Research (COREQ) checklist to enhance transparency and rigor in the reporting of qualitative findings (Table S1) [25].

### 2.3. Participant Recruitment

Participants were recruited using purposive sampling. Potential participants were selected to ensure variation in demographic characteristics, social media use, and experiences seeking information about dental implant treatment. Although the eligibility criteria included individuals seeking implant and/or prosthodontic treatment, all participants included in the final sample were candidates for dental implant treatment. Participants had already undergone clinical assessment in the periodontics clinics and had been identified as suitable candidates for dental implant treatment before being approached for participation. Information regarding individuals who declined participation and their reasons for non-participation was not systematically recorded during recruitment.

Recruitment was conducted in dental care settings, where potential participants were approached face-to-face and provided with information about the study.

Eligible participants included individuals who: (a) were aged 18 years or older and were currently seeking or undergoing dental implant and/or prosthodontic treatment; (b) were able to communicate in Arabic or English; (c) reported using at least one social media or online platform to access information; and (d) were willing to participate in an audio-recorded interview lasting up to 30 min. Individuals who were unable to provide informed consent or who experienced difficulties participating in an interview were excluded.

Potential participants received a participant information sheet explaining the study objectives, procedures, confidentiality measures, and voluntary nature of participation. Written informed consent was obtained prior to data collection, and interviews were scheduled at times convenient to participants.

### 2.4. Data Collection

Interviews were conducted by two researchers (W.A., female), a qualified dentist and family dentistry master’s student, and (R.S., male), a clinical consultant and faculty member at a dental school. Both interviewers had previous experience in qualitative research and shared research interests in oral health, digital health communication, and public health.

Data collection took place between 1 March 2026 and 30 July 2026. Interviews were conducted in private areas within the dental school, including vacant dental clinics and faculty offices, to ensure participant privacy and minimize interruptions. No individuals other than the participant and interviewer were present during the interviews. Before each interview, participants were informed about the study objectives, the researchers’ professional backgrounds, and the voluntary nature of participation.

Data were generated through one-to-one semi-structured interviews using an interview guide developed by the research team. The semi-structured interview guide was developed by the research team based on the study objectives and a review of the literature on health misinformation, information credibility, and social media use, following recommendations for qualitative interview guide development [26]. Prior to data collection, the interview guide was pilot tested with two individuals from the target population to assess the clarity, relevance, and flow of the questions. Minor revisions were made to improve wording and sequencing of the interview prompts. The guide covered social media exposure, credibility assessment, experiences with misinformation, information verification practices, treatment decision-making, trust in information sources, and recommendations for improving patient education. Open-ended questions and follow-up prompts were used to facilitate in-depth discussion. A summary of the interview topics is provided in Table 1.

Interviews were conducted in Arabic, lasted approximately 30 min, and were audio-recorded with participants’ consent. Audio recordings were transcribed verbatim and anonymized prior to analysis. The transcripts were then translated into English by bilingual members of the research team. To preserve linguistic and cultural meaning, translated transcripts were reviewed and compared with the original Arabic transcripts, and any ambiguities were discussed among the researchers before analysis.

Recruitment and interviewing continued until no substantial new insights relevant to the study objectives were being generated and sufficient depth and diversity of perspectives had been achieved [27]. The interviewers had no prior personal or professional relationship with the participants before study recruitment. Any interaction occurred only for the purposes of recruitment and participation in the study.

### 2.5. Data Analysis

Data were analyzed using Braun and Clarke’s reflexive thematic analysis (RTA) approach [28]. Data analysis was undertaken by W.A., R.S., and M.A., who were actively involved in coding, theme development, and interpretation of the findings.

Consistent with Braun and Clarke’s reflexive thematic analysis, coding agreement or intercoder reliability was not assessed. Instead, coding, theme development, and interpretation were informed through ongoing discussions among the researchers, which supported reflexive engagement with the data and refinement of developing interpretations.

All interview recordings were transcribed verbatim, anonymized, and reviewed repeatedly to facilitate familiarization with the dataset. During this stage, the researchers immersed themselves in the data and documented initial observations relevant to the study aims.

An inductive coding approach was adopted, allowing codes to be developed from participants’ accounts rather than from a pre-existing theoretical framework. Initial codes were generated inductively through close engagement with the transcripts and recorded within a developing codebook. The codebook served as an evolving organizational tool rather than a predetermined coding framework and was used to document code definitions, coded extracts, and analytic notes throughout the process [29]. Codes were continuously reviewed, refined, combined, or separated as researchers moved between the transcripts and developing interpretations. Related codes were subsequently organized into candidate themes. These themes were reviewed against both the coded data and the full dataset and further developed to ensure they captured meaningful patterns relevant to the study objectives.

NVivo software (Lumivero, version 15, Denver, CO, USA) was used to assist with data management, coding, and organization. The software supported the analytic process, while interpretation and theme development remained the responsibility of the research team.

Analysis was iterative and involved continual movement between the data, codes, codebook, and developing themes. Codes reflecting similar meanings were grouped together and examined for broader patterns across participants’ accounts. Themes were iteratively refined to ensure they meaningfully captured patterns relevant to the study objectives. Regular discussions among the research team supported reflexive engagement with the data, critical examination of interpretations, and refinement of the thematic framework. Reflexivity was maintained throughout the study by encouraging researchers to reflect on how their professional backgrounds, experiences, and assumptions might influence data interpretation. The research team regularly discussed coding decisions, theme development, and alternative interpretations to promote critical engagement with the data and enhance analytical transparency. Consistent with reflexive thematic analysis, recruitment ceased when the dataset was considered adequate to address the study aims [28]. Data collection and analysis occurred concurrently, with recruitment continuing until the research team determined that sufficient depth, richness, and diversity of perspectives had been obtained to address the study objectives. This decision was informed by ongoing review of interview data and developing themes, which indicated that subsequent interviews were largely reinforcing existing patterns rather than providing substantial new insights relevant to the research questions. Consistent with reflexive thematic analysis, the decision to stop recruitment was based on the adequacy of the dataset to address the study aims rather than on achieving statistical representation. Sample adequacy was judged according to the richness, depth, and relevance of the dataset in relation to the study objectives. The interviews generated detailed and information-rich accounts across all areas addressed in the interview guide, providing sufficient depth to support meaningful interpretation of participants’ experiences and perspectives. The final sample provided sufficient diversity of perspectives and detailed accounts to support meaningful interpretation of participants’ experiences and information-evaluation practices.

Anonymized participant quotations are presented to illustrate and support the final themes, with participants identified using study codes (e.g., P1, P2) to maintain confidentiality.

## 3. Results

### 3.1. Participants Characteristics

Sixteen participants who were candidates for dental implant treatment participated in the study. Participants ranged in age from 24 to 56 years (mean age = 38.5 years) and included 11 males (68.8%) and 5 females (31.3%). Educational backgrounds varied from primary school to bachelor’s-level education. Participants reported using a wide range of online platforms, including WhatsApp, Snapchat, TikTok, Facebook, Instagram, YouTube, X (formerly Twitter), and Google to access information. Participant characteristics are summarized in Table 2.

Five themes were identified that captured how participants encountered, evaluated, and responded to information about dental implant treatment on social media. The themes reflected participants’ trust in information sources, verification practices, use of social and professional influences, responses to potentially misleading content, and the role of social media in treatment-related decision-making.

### 3.2. Themes

#### 3.2.1. Theme 1. Trust Shaped by Professional Credibility and Personal Experience

Trust played an important role in how participants evaluated information about dental implant treatment. Most participants did not rely on online information alone. Instead, they turned to dentists, trusted organizations, and personal experiences to decide whether information was trustworthy.

1.1 Trust in Dentists and Professional Expertise

Dentists were consistently identified as the most trusted source of information. Although participants often used social media and internet searches to learn about dental implants, online information was generally viewed as a starting point rather than a basis for decision-making. Many believed that dentists could provide accurate information because of their professional expertise and understanding of individual patient needs. As Participant 13 explained:

*“I’d contact my dentist first. I might search Google as well, but anyone can post information online. My dentist is someone I know and trust.”*

(P13)

Participants also emphasized the value of personalized advice. One participant explained:

*“My dentist understands my situation and knows my condition. Social media doesn’t know what I’m dealing with.”*

(P9)

1.2 Trust in Official and Government Sources

In addition to dentists, participants described trusting official health organizations, particularly the Ministry of Health. Government sources were often viewed as credible because they were perceived to provide information that had been reviewed and validated before publication. As Participant 14 explained:

*“I trust the Ministry of Health because I know who they are, whereas I may not know who is behind information posted online.”*

(P14)

Similarly, Participant 8 stated:

*“Government organizations, definitely. They usually don’t publish information unless it’s been carefully reviewed first.”*

(P8)

Although official sources were generally viewed as trustworthy, participants still tended to prioritize advice from their own dentists when making treatment decisions.

1.3 Trust Shaped by Personal Experience

Trust was also shaped by previous experiences with dental care. Participants often described feeling more confident in dentists they had visited before and with whom they had established positive relationships over time. Familiarity with a provider and previous treatment experiences gave participants greater confidence in the information and recommendations they received.

As Participant 6 explained:

*“People recommend certain dentists because they’ve had good experiences with them. They feel comfortable with them and keep going back for years.”*

(P6)

Trust was often reinforced through ongoing relationships with providers. As Participant 8 noted:

*“Once trust is established between me and the dentist, that’s enough.”*

(P8)

For many participants, trust was shaped not only by professional qualifications but also by familiarity, previous experiences, and long-term relationships with dental providers.

#### 3.2.2. Theme 2. Verifying Information Before Making Decisions

Participants rarely accepted implant-related information at face value. Instead, they often checked information by consulting dentists, comparing sources, and reviewing patient experiences before making treatment decisions.

2.1 Seeking Professional Confirmation

Many participants described consulting dental professionals before acting on information encountered online. Rather than relying solely on social media or internet searches, they often turned to dentists or specialists to determine whether information was accurate and relevant to their individual circumstances. As Participant 6 explained:

*“Honestly, I ask people who specialize in that area. If it’s not my field, I consult them and ask whether it’s true and whether it’s suitable for me.”*

(P6)

Professional advice became particularly important when participants encountered conflicting information. In such situations, they often sought guidance from specialists with relevant expertise.

2.2 Cross-Checking Multiple Sources

Many participants described comparing information from different sources rather than relying on a single source. Cross-checking helped them identify inconsistencies, confirm information, and feel more confident about treatment-related decisions. As Participant 4 explained:

*“I don’t rely on one video. I watch several videos from different dentists.”*

(P4)

Similarly, Participant 15 described using a combination of online resources and personal networks when professional advice was not immediately available:

*“If a doctor isn’t available, then I’d search and compare different sources on Google and ask people around me.”*

(P15)

For these participants, consistency across sources appeared to increase confidence in the accuracy of information and reduce uncertainty before acting upon it.

2.3 Using Reviews, Comments, and Patient Experiences

In addition to consulting professionals and comparing sources, many participants relied on online reviews, comments, and patient experiences when evaluating treatment information. Such feedback was often viewed as more authentic than promotional content because it reflected real treatment experiences. As Participant 11 explained:

*“I usually check Google Maps reviews and pay attention to what people are complaining about.”*

(P11)

Similarly, Participant 12 described using comments to learn from other people’s experiences:

*“I read the comments because people share their experiences, whether something worked for them or not.”*

(P12)

Participants often viewed reviews, comments, and patient experiences as practical ways of judging credibility and reducing uncertainty before making treatment decisions.

2.4 Emerging Digital Verification Strategies

A small number of participants reported using artificial intelligence (AI) tools as part of their verification process. Platforms such as ChatGPT, Gemini, and Meta AI were used to obtain additional perspectives and compare information from different sources.

As Participant 9 explained:

*“When I see information online, I don’t rely on one source. I usually look it up on ChatGPT or Gemini and compare what they say.”*

(P9)

Similarly, Participant 14 stated:

*“I’d ask Meta AI and compare its answer with the information I’d already found.”*

(P14)

Although only a few participants mentioned these tools, their accounts suggest that AI is beginning to play a role in how some patients evaluate health information. Rather than replacing professional advice, these tools appeared to be incorporated into a broader verification process that also included professional consultation, online searches, and patient experiences.

#### 3.2.3. Theme 3. Reputation and Social Proof Shape Treatment Choices

Participants often relied on recommendations, patient experiences, online reviews, and treatment outcomes when assessing dentists and making treatment decisions. For many, these forms of social proof helped reduce uncertainty.

3.1 Recommendations From Family and Friends

Recommendations from family members, friends, and acquaintances played an important role in how participants selected dentists and evaluated treatment options. Many relied on recommendations from people they knew because these reflected direct experiences rather than advertising. These recommendations acted as a form of social proof, allowing participants to draw on the experiences of others when evaluating providers and making treatment-related decisions.

As Participant 7 explained:

*“If someone close to me had been treated by that dentist and had a good experience, I’d feel much more confident about going to them.”*

(P7)

Similarly, Participant 14 stated:

*“I’d only trust them if I already knew the dentist, or if they were recommended by people I trust.”*

(P14)

3.2. Patient Experiences as Evidence of Quality

Beyond recommendations from family and friends, participants often relied on the experiences of previous patients when evaluating dentists and treatment options. First-hand accounts were frequently viewed as more convincing than promotional content because they reflected real treatment outcomes.

As Participant 16 explained:

*“Hearing it from someone who has actually experienced it is much more convincing.”*

(P16)

Participant 7 expressed a similar view:

*“I’d rather hear from someone who has actually tried the treatment than just listen to marketing.”*

(P7)

Many participants viewed patient experiences as a practical form of evidence, particularly when several individuals reported similar experiences. Such accounts helped them judge the quality of a dentist and gain confidence in treatment recommendations.

3.3 Online Presence and Visible Clinical Work

Participants also described using a dentist’s online presence to assess credibility. Rather than relying solely on claims or advertisements, many wanted to see examples of clinical work and treatment outcomes.

Participant 6 noted:

*“When I see dentists posting their work on their accounts, I feel more comfortable. If they weren’t confident in their work, they wouldn’t post it.”*

(P6)

#### 3.2.4. Theme 4. Navigating Misinformation Through Skepticism and Critical Judgment

Participants were often skeptical of information encountered on social media. Rather than accepting claims at face value, they relied on common sense, personal experience, and professional advice to judge whether information was trustworthy.

4.1 Distrust of Advertisements and Commercial Motives

Many participants were cautious about advertisements because they felt that their primary purpose was to attract patients rather than provide balanced information. Suspicion was particularly common when claims appeared overly positive or when prices seemed unrealistic.

Participant 16 expressed skepticism toward promotional content:

*“Most of the dental ads I see are from clinics or dental practices and, to be honest, I don’t really trust them.”*

(P16)

Similarly, Participant 7 believed that financial motives often influenced the information being shared:

*“Some of them seem more interested in making money.”*

(P7)

Several participants felt that advertisements often exaggerated treatment benefits. When promotional claims did not match actual treatment experiences, participants became more cautious about trusting online information.

4.2 Identifying Exaggerated and Unrealistic Claims

Participants frequently questioned claims that seemed too good to be true, particularly those promising pain-free treatment, immediate results, or guaranteed success. Such claims often increased skepticism rather than confidence.

As Participant 6 explained:

*“I’ve seen plenty of ads like that, but I’ve never really believed them. You can’t have surgery done in five minutes and expect it to be completely painless.”*

(P6)

A similar view was expressed by Participant 13:

*“Definitely not. Even replacing one tooth takes time. Implant treatment usually takes months.”*

(P13)

Several participants felt that these claims were rarely explained properly. As a result, exaggerated promises often reduced trust and encouraged them to look for more information before believing them.

4.3 Recognizing Personal Responsibility in Evaluating Information

Many participants felt that it was their responsibility to evaluate information carefully before acting on it. Even when information came from a familiar source, they believed it was important to consider where it came from and whether it was credible.

Participant 4 explained:

*“People should look for the right information and not believe the first advertisement they see.”*

(P4)

Similarly, Participant 15 stated:

*“Whenever I come across new information, I think it’s important to check where it’s coming from.”*

(P15)

Several participants also emphasized the role of personal judgment. For example, Participant 13 explained:

*“You have to use your common sense. Most of the time, you can tell when something just doesn’t sound right.”*

(P13)

#### 3.2.5. Theme 5. Social Media as a Source of Information, Not the Final Authority

Participants commonly used social media to learn about dental implant treatment. However, they generally viewed it as a starting point rather than a basis for treatment decisions.

5.1 Learning About Procedures and Treatment Options

Many participants used social media to learn how dental implant treatment is performed and what to expect from the procedure. This helped them become more familiar with the treatment process before seeking professional advice.

As Participant 9 explained:

*“I searched for how dental implants are done, and the first thing I found explained that they place a screw and then attach the tooth.”*

(P9)

The participant added:

*“It helped me understand how the implant is placed.”*

(P9)

5.2 Visual Content Facilitates Understanding

Visual content played an important role in helping participants understand dental procedures. Videos, clinical cases, and before-and-after images were often viewed as easier to understand than written explanations.

As Participant 9 explained:

*“It was a video showing the procedure.”*

(P9)

Similarly, Participant 5 stated:

*“They show the results before and after treatment and talk about what they did.”*

(P5)

These accounts suggest that visual content helped participants better understand treatment procedures and outcomes by making information more concrete and accessible.

5.3 Professional Consultation Remains Essential

Although social media was valued as a source of information, participants generally viewed professional consultation as an essential part of treatment-related decision-making.

Participant 13 stated:

*“Any other source is less trustworthy. Even if it’s from an official organization, I’d still check with my dentist first.”*

(P13)

These responses suggest that participants valued social media as a source of information, but not as a substitute for professional consultation.

5.4 Expectations for Professional Education and Public Awareness

Many participants felt that dentists and health organizations should play a more active role in educating the public and providing reliable information online.

As Participant 9 stated:

*“They should post information on social media and run awareness campaigns.”*

(P9)

Similarly, Participant 15 emphasized:

*“Increase public education and media coverage.”*

(P15)

Participants therefore viewed healthcare professionals not only as trusted information sources but also as having an important role in improving the quality of information available online.

## 4. Discussion

This study explored how patients evaluate and respond to dental implant information encountered on social media. Overall, participants viewed social media as a useful source of information but not as a substitute for professional advice. Trust in dentists and official sources, active verification behaviors, reliance on patient experiences and reputation, and skepticism toward promotional claims emerged as important strategies that participants reported using to navigate online information and support informed decision-making.

The findings of Theme 1, Trust in Dentists and Professional Expertise, highlight the central role of trust in how participants evaluated information about dental implant treatment. Although social media was commonly used to obtain information, dentists remained the most trusted source when treatment decisions were involved. Participants often relied on their dentists because of previous experiences, established relationships, and confidence in their expertise. This finding is consistent with previous research showing that trust is a key component of the dentist–patient relationship and influences treatment decisions [30,31,32].

Trust also extended beyond professional qualifications alone. Participants emphasized the importance of familiarity, previous interactions, and recommendations from trusted individuals. Official health organizations were generally viewed as credible sources, although advice from a personal dentist was often given greater weight. Overall, trust appeared to be an important foundation for evaluating online information and making decisions about dental implant treatment.

The findings of Theme 2, Verifying Information Before Making Decisions, suggest that participants rarely accepted information about dental implants without checking it first. Instead, they described consulting dentists, comparing different sources, reviewing patient experiences, and seeking clarification when information appeared uncertain. These self-reported behaviors may support resilience to misinformation and reflect several important aspects of digital health literacy, including information evaluation, source verification, and informed decision-making [23,33,34].

Participants used a range of strategies to verify information. Many sought professional confirmation, particularly when information conflicted with other sources. Others compared information across platforms, while some reported using AI tools such as ChatGPT, Gemini, and Meta AI to obtain additional perspectives. Although the use of AI for evaluating dental information remains under investigation, recent studies suggest that these technologies are increasingly being explored as sources of health information despite concerns regarding accuracy and the need for professional oversight [35]. Similarly, AI-generated responses may be perceived as authoritative or credible despite the potential for incomplete, inaccurate, outdated, or non-contextualized information. While such tools may support information seeking and improve access to information, they do not replace professional clinical judgment and may generate responses that are inconsistent with current evidence [35]. Consequently, information obtained from AI platforms should be interpreted cautiously and considered alongside professional advice and evidence-based sources. These findings highlight the active role patients may play in evaluating implant-related information before making treatment-related decisions.

The findings of Theme 3, Reputation and Social Proof Shape Treatment Choices, suggest that participants frequently relied on the experiences and opinions of others when evaluating dentists and making decisions about dental implant treatment. Recommendations from family, friends, and previous patients were often viewed as credible and useful sources of information, particularly when participants felt unable to judge clinical information themselves. Reputation therefore became an important way of identifying trustworthy providers and gaining confidence in treatment decisions, consistent with previous research highlighting the influence of recommendations and positive endorsements on patient trust and treatment choices [30].

Participants also valued patient experiences, online reviews, and visible treatment outcomes. First-hand accounts were often viewed as more authentic than promotional content and helped participants judge the quality of care. Similar findings have been reported in studies showing that online reviews influence perceptions of dental care quality, trust, and willingness to seek treatment [36,37]. Although social proof may help patients navigate treatment decisions, it may also reflect personal experiences rather than clinical evidence. Patient testimonials and online reviews may provide useful insight into individual experiences; however, they do not necessarily reflect treatment quality, clinical effectiveness, or evidence-based practice. Personal experiences can be influenced by individual expectations, treatment outcomes, and subjective interpretations, and may therefore not provide an objective basis for evaluating treatment options. As such, reviews, recommendations, and patient experiences were often used alongside, rather than in place of, professional advice.

The findings of Theme 4, Navigating Misinformation Through Skepticism and Critical Judgment, suggest that participants did not automatically trust information encountered on social media. Many questioned claims that seemed unrealistic, particularly those promising pain-free treatment, immediate results, or guaranteed outcomes. This cautious approach may represent an important behavior that supports resilience to misinformation and is consistent with concerns that social media may create unrealistic expectations by presenting simplified or overly positive information about implant treatment [3,38].

Participants were also cautious of content they perceived as commercially motivated. This finding aligns with research showing that much of the implant-related content on social media is promotional in nature and often lacks an evidence-based focus [39]. Similar concerns have been reported for implant practice websites, where information about complications and treatment limitations is frequently limited or absent [40].

The findings of Theme 5, Social Media as a Source of Information but Not the Final Authority, suggest that participants mainly used social media to learn about dental implant treatment rather than to make treatment decisions. Many used online platforms to understand procedures, explore treatment options, and develop expectations about treatment. This supports previous research showing that digital technologies can improve patient knowledge and access to oral health information [41,42].

However, participants did not view social media as a replacement for professional advice. Instead, online information was often used as a starting point before consulting a dentist. Similar findings have been reported among individuals who use online health information to improve their understanding while continuing to rely on healthcare professionals when making personal health decisions [43]. This may be particularly relevant in implant dentistry, where treatment expectations can be shaped by information encountered online [3].

Taken together, the findings suggest that information seeking, trust, verification, social influences, and critical evaluation may contribute to resilience against dental implant misinformation. However, these findings reflect participants’ reported practices and perceptions rather than direct evidence of their ability to identify or resist misinformation.

**Figure 1:**
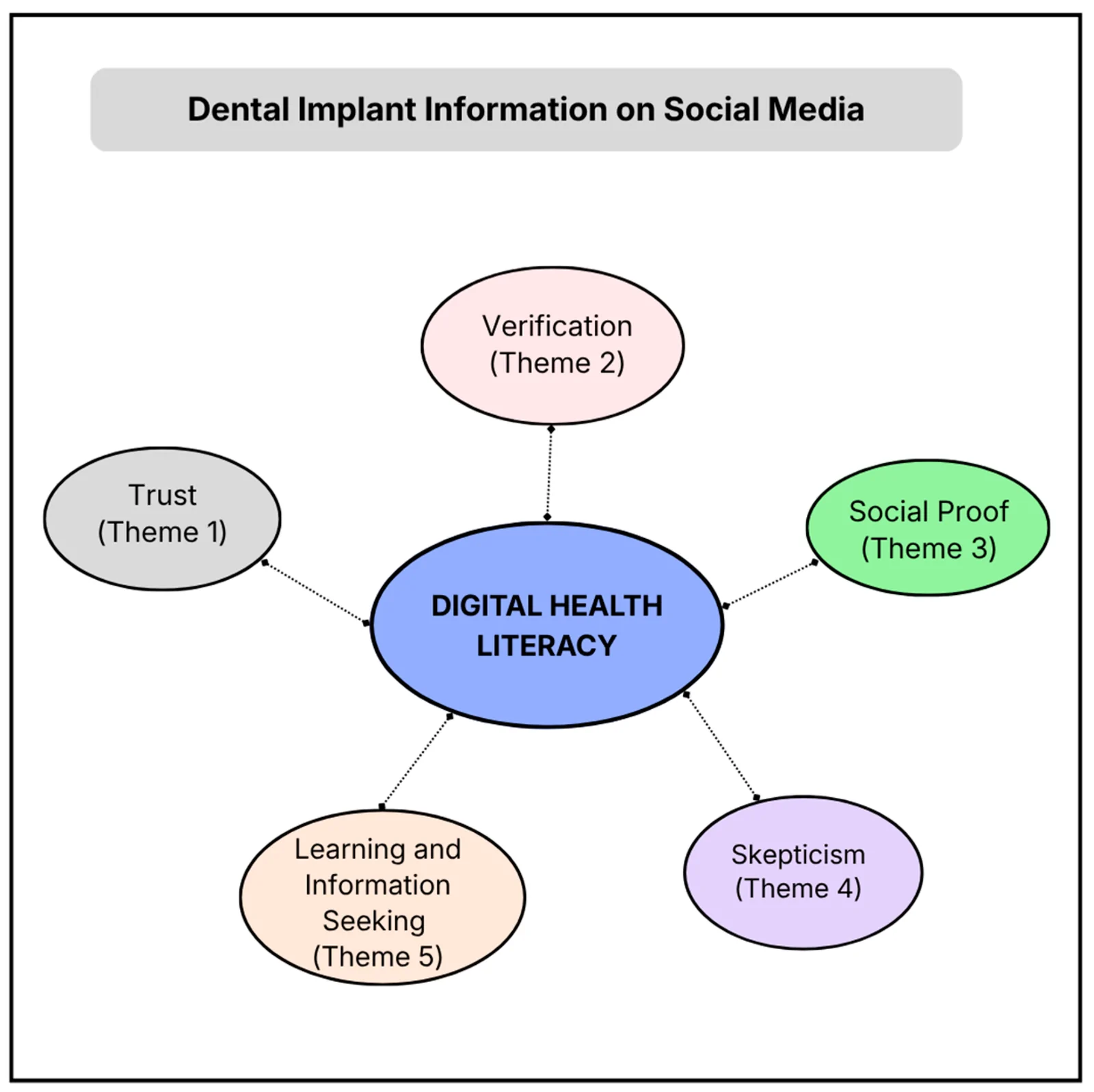
Conceptual framework illustrating an interpretive synthesis of the themes identified in participants’ accounts of evaluating dental implant information on social media. The framework highlights themes related to trust, verification, social proof, skepticism, and information seeking and should not be interpreted as a tested causal pathway.

While the present study did not directly assess participants’ ability to distinguish accurate information from misinformation, it identified several behaviors that may help patients evaluate online information and respond to potentially misleading claims. Participants did not rely solely on information encountered online; instead, they drew on multiple strategies to assess credibility before making treatment decisions. Figure 1 presents a conceptual framework illustrating an interpretive synthesis of the themes identified in the interviews. The framework highlights how trust, verification, social proof, skepticism, and information-seeking behaviors were reflected in participants’ accounts of evaluating dental implant information encountered on social media. Digital health literacy is presented as a central concept within this context. The framework represents an interpretive summary of the findings and should not be interpreted as a tested causal model or as evidence of direct relationships among the identified themes.

### 4.1. Implications for Health Literacy and Clinical Practice

The findings suggest that health literacy initiatives in dentistry should focus not only on improving access to information but also on helping patients evaluate the credibility of online content. Practical strategies may include encouraging patients to verify information through multiple sources, consult qualified dental professionals, and consider whether information is supported by evidence rather than personal opinion or promotional claims.

The findings also highlight opportunities for dentists, healthcare organizations, and content creators to improve the quality of dental information available online. Providing clear, accessible, and evidence-based content, including explanations of treatment benefits, risks, limitations, and expected outcomes, may help patients make more informed decisions. Professional dental organizations may also play an important role by developing reliable educational resources and increasing their visibility on social media platforms.

Finally, social media platforms and policymakers may help support informed decision-making by promoting credible health content, improving access to information from verified healthcare sources, and encouraging greater transparency regarding promotional and sponsored dental content. Together, these approaches may support digital health literacy and help patients navigate dental information more effectively in online environments.

### 4.2. Strengths and Limitations

This study has several strengths. To our knowledge, it is among the first qualitative studies to explore how patients evaluate and respond to dental implant information encountered on social media. The use of in-depth interviews provided rich insights into how patients assess information, verify claims, and make treatment-related decisions. In addition, the study offers a patient-centered perspective on misinformation resilience, a topic that has received limited attention within dental research.

Several limitations should be considered. The study was conducted in a single country and involved participants recruited from one clinical setting, which may limit the transferability of the findings to other populations and healthcare contexts. In addition, all participants were candidates for dental implant treatment and had experience seeking treatment-related information, which may have influenced how they engaged with and evaluated online content. Their perspectives may also differ from those of individuals who rarely use digital sources when making healthcare decisions.

The findings are also based on self-reported experiences and perceptions and may therefore be influenced by recall or social desirability bias. As with qualitative research generally, the aim was to gain an in-depth understanding of participants’ experiences rather than produce statistically generalizable findings. Future research involving larger and more diverse populations could further explore how patients evaluate online dental information and respond to misinformation across different settings.

## 5. Conclusions:

- Trust was central to information evaluation. Dentists remained the most trusted source of information, even among participants who frequently used social media.
- Participants actively verified information before making treatment decisions. Common strategies included consulting dentists, comparing multiple sources, and reviewing patient experiences.
- Reputation and social proof influenced treatment choices. Recommendations, online reviews, and patient experiences were commonly used to assess the credibility of dentists and treatment information.
- The identified behaviors should be viewed as potential contributors to misinformation resilience rather than direct evidence of misinformation resilience. Trust, verification, social influences, and critical evaluation may help patients navigate inaccurate or misleading information encountered online.

## Figures and Tables

**Table 1:** Summary of interview topic guide questions.

Interview Topic	Example Questions/Prompts
Social media use and exposure to dental information	How often do you encounter information about dental implants or prosthodontic treatment on social media? Prompts: Which platforms do you use most? What types of content do you typically see?
Perceptions of information credibility	How do you decide whether online dental information is trustworthy? Prompts: What factors influence your confidence in a source? How the source of information affect your trust?
Experiences with questionable or conflicting information	Have you ever encountered information about dental implants or prosthodontic treatment that you later questioned or thought might be inaccurate? Prompts: How did you evaluate the information?
Responses to uncertain information	How do you react when you encounter information that you are unsure about? Prompts: How conflicting information could affect your understanding, confidence, or decision-making?
Information verification practices	What do you usually do when you want to confirm whether information is accurate? Prompts: How do you seek dental professional advice?
Influence on treatment-related decisions	How information encountered on social media influenced your perceptions, treatment preferences, or decisions regarding dental implants or prosthodontic treatment?
Role of family, friends, and social networks	To what extent do family members, friends, or other patients influence your views about dental treatment? Prompts: How do their opinions compare with professional advice?
Trust in professional and official sources	How do you compare information provided by dentists, social media users, and other online sources? Prompts: How do you accept/reject information provided by these sources?
Confidence in evaluating online information	How confident are you in determining whether online dental information is accurate? Prompts: What factors make this easier or more difficult?
Recommendations for improving patient education	What could dentists, healthcare organizations, or social media platforms do to help people make informed decisions about dental treatment information encountered online?

**Table 2:** Participant characteristics (N = 16).

Characteristic	n (%)
**Gender**	
Male	11 (68.8)
Female	5 (31.3)
**Age group (years)**	
20–29	7 (43.8)
30–39	2 (12.5)
40–49	4 (25.0)
≥50	3 (18.8)
**Educational level**	
Primary school	4 (25.0)
High school	4 (25.0)
Diploma	1 (6.3)
Bachelor’s degree or higher	7 (43.8)
**Occupation**	
Housewife	5 (31.3)
Employed	11 (68.8)
**Most frequently reported online/social media platforms**	
WhatsApp	7 (43.8)
Snapchat	8 (50.0)
TikTok	8 (50.0)
Facebook	6 (37.5)
Instagram	5 (31.3)
YouTube	5 (31.3)
X (Twitter)	2 (12.5)
Google/Search engines	7 (43.8)

## Data Availability

The data presented in this study are available on request from the corresponding author, the data are not publicly available due to ethical and confidentiality considerations.

## Supplementary Materials

The following supporting information can be downloaded at https://www.mdpi.com/article/10.3390/healthcare14193298/s1, Table S1 Consolidated criteria for reporting qualitative studies (COREQ): 32-item checklist.

## Abbreviations

The following abbreviations are used in this manuscript:

AI	Artificial Intelligence
COREQ	Consolidated Criteria for Reporting Qualitative Research
IRRB	Institutional Research Review Board
MOH	Ministry of Health
RTA	Reflexive Thematic Analysis
